# The effect of illness perception on psychosocial adjustment of patients with breast cancer and their spouses: actor–partner independence model

**DOI:** 10.1186/s40359-024-01741-6

**Published:** 2024-05-29

**Authors:** Yan-feng Wang, An-kang Liu, Jin-zhen Dai, Ji-ping Zhang, Hui-hua Chen, Xiao-hao Jiang, Lu Tang, Yong-yue He, Qiao-hong Yang

**Affiliations:** 1 School of Nursing, Jinan University, Room 528, Guangzhou City, Guangdong Province China; 2 The First Affiliated Hospital of Jinan University, Guangzhou City, Guangdong Province China; 3 Shanwei Second People’s Hospital, Shanwei City, Guangdong Province China

**Keywords:** Breast cancer, Patient, Spouse, Illness perception, Psychosocial adjustment to illness

## Abstract

**Objective:**

With the increase in the prevalence rate and improvements in the survival of breast cancer patients, there is a growing interest in understanding the level of psychosocial adjustment in these patients. The study aimed to describe the illness perception and psychosocial adjustment levels of both breast cancer patients and their spouses, to use the Actor-Partner Interdependence Model (APIM) to clarify the actor-partner relationships between spouses, and to explore the impact of illness perception on psychosocial adjustment to the disease within the joint actions of both spouses.

**Methods:**

A total of 216 female patients with breast cancer and their spouses participated in the study. They were selected from two tertiary hospitals in Guangdong Province, China from October 2022 to May 2023 using a convenience sampling method. The participants were assessed using the Brief Illness Perception Questionnaire and the Psychosocial Adjustment to Illness Scale to examine the relationship between illness perception and psychosocial adjustment. AMOS24.0 was used to test and analyze the actor-partner interdependence model.

**Results:**

The illness perception score (57.75 ± 10.91) was slightly higher than that of the spouse (57.10 ± 11.00), and the psychosocial adjustment score (64.67 ± 6.33) was slightly lower than that of the spouse (64.76 ± 7.49). The results of the actor-partner interdependence model indicated that there was a couple partner between breast cancer patients and their spouses: the spouse’s illness perception significantly affected the patient’s psychosocial adjustment (*β* = 0.095, *p* = 0.015); the patient’s illness perception also significantly affected the spouse’s psychosocial adjustment (*β* = 0.106, *p* = 0.033). Among them, the patient’s psychosocial adjustment was found to be related to the patient’s illness comprehensibility or coherence of illness (*β* = 0.433, *p* = 0.009), the spouse’s emotional illness representation (*β* = 0.218, *p* = 0.037), and the spouse’s illness comprehensibility or coherence of illness (*β* = 0.416, *p* = 0.007), while the spouse’s psychosocial adjustment was only related to the spouse’s illness comprehensibility or coherence of illness (β = 0.528, *p* = 0.007).

**Conclusions:**

The psychosocial adjustment of breast cancer patients is affected by both their own and spouse’s illness perception. Therefore, in the future, the healthcare staff can implement early psychological interventions for patients diagnosed with breast cancer and their spouses as a unit to promote the psychosocial adjustment of them.

## Background

Breast cancer (BC) is the most common malignancy threatening women’s health worldwide. According to the Global Cancer Statistics Report [1], there are approximately 2.26 million new cases of BC each year, ranking first in the incidence of cancer. In China, there are approximately 368,000 new cases of BC annually, and the number is increasing yearly [2]. With advancements in medicine, the survival rate of patients with BC has gradually increased. In the United States, the five-year survival rate of patients with early-stage BC is 90% [3], and in China, the five-year survival rate of patients with middle- and advanced-stage BC has also increased from 20 to 60% [4]. With the prolongation of the survival cycle of BC patients, patients faced not only various problems arising from disease treatment and symptom management [5], but also complex physical and psychological changes that affect the quality of life [6, 7].

Psychosocial adjustment is an essential facet of enhancing the quality of life for patients [8]. It refers to the ability of patients with BC to adapt and coordinate in multiple aspects [9], such as family life, social life, and sex, when they endure various symptoms and pain caused by the diagnosis and treatment of BC. A study has shown that illness perception is a pivotal predictor of the physical and mental health of patients and can significantly affect their psychosocial adjustment level [10]. Several theories and models have highlighted the profound impact of patients’ beliefs and perceptions of their disease and symptoms on their psychological adjustment [11, 12]. In addition, Leventhal’s self-regulatory model suggests that when people are stimulated (disease diagnosis or symptom presentation), they form an illness perception [13]. Illness perception [14] (IP) refers to the process in which patients adjust, modify, and cope with disease cognition and emotional response based on previous disease experience when facing a disease or health threat, including the cognitive representation (problem-centred and different responses to the individual’s perception of the disease), emotional representation (pmotionally centered, tending to adopt a certain emotion to cope), and illness comprehensibility (degree of knowledge of the disease).

Cancer, as a kind of “we disease”, not only affects the physical and mental health of patients with BC but also introduces challenges and pressure to the entire family [15]. Particularly in intimate relationships, there exists a strong interaction between two individuals [16], and an individual’s emotion can be easily conveyed between the two [17]. During the diagnosis and treatment of patients with BC, the spouse, as their primary caregiver and source of emotional and financial support also suffers from different levels of stress, which are similar to or even stronger than that of the patient [18]. However, the majority of current studies only discuss the influence of patients’ illness perception on the psychosocial adjustment of illness, neglecting the influence of patients and their spouses on the psychosocial adjustment to illness (PAI).

**Fig. 1:**
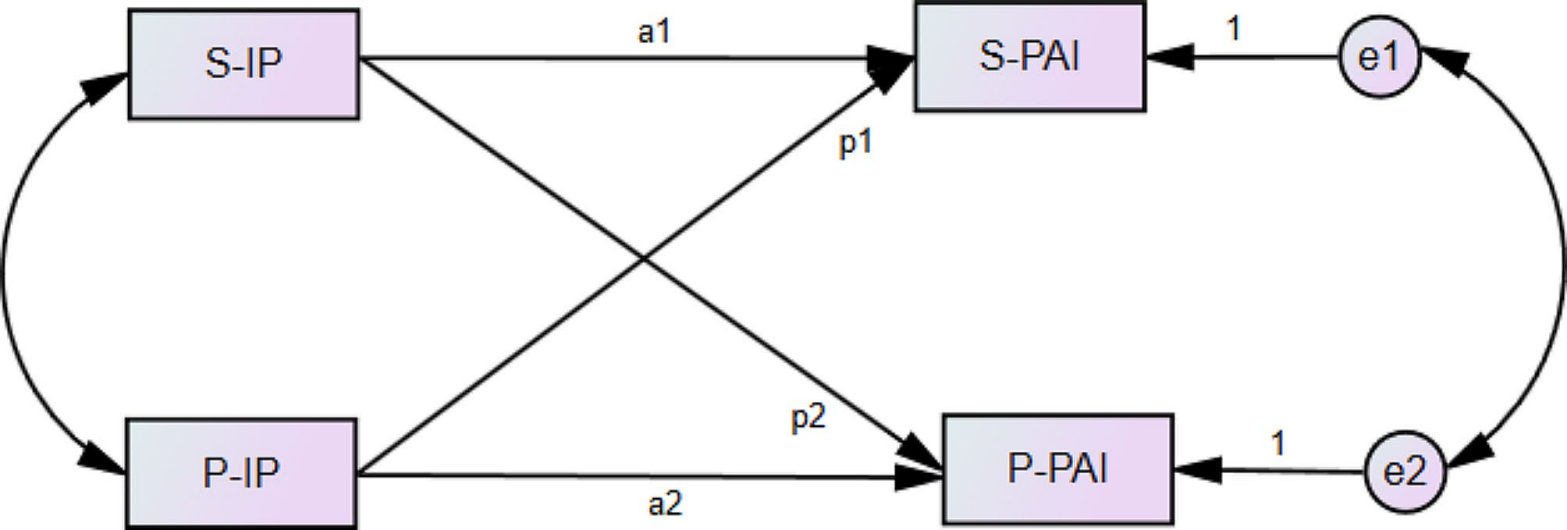
The hypothesis framework of IP and PAI between patients and spouses. *Note.* S-IP represents Spouse-Illness Perception; P-IP stands for Patient-Illness Perception; P-PAI represents Patient-Psychosocial Adjustment to Illness; S-PAI refers to Spouse-Psychosocial Adjustmentto Illness. a1: S-IP → S-PAI; a2: P-IP → P-PAI; p1:P-IP → S-PAI; p2: S-IP → P-PAI; where k equals the ratio of patient to spouse (p/a)

Therefore, this study aims to explore the impact of disease perception on disease psychosocial adjustment under the joint action of husband and wife by describing the illness perception and psychosocial adjustment level of BC patients and their spouses, and using APIM to clarify the actor-partner relationship between husband and wife. The hypothesis framework of this study is shown in Fig. 1.

## Method

### Design and participants

A cross-sectional design using a survey was employed in this study. Patients with BC and their spouses from two tertiary hospitals in Guangdong Province, China, were selected between October 2022 and May 2023 using a convenience sampling method. Inclusion criteria for patients were: (1) diagnosis of primary BC (without metastatic); (2) having normal cognitive and understanding abilities; (3) being aware of BC diagnosis and participating in this study voluntarily. Exclusion criteria for patients were: (1) previous diagnoses of other primary malignancies; (2) a state of illness that does not permit collaboration with the investigator. Inclusion criteria for spouses were: (1) being the legal spouse of patients with BC; (2) having normal cognitive and understanding abilities; (3) knowing the patient’s BC diagnosis and participated in this study voluntarily. Exclusion criteria for spouses were: (1) having a serious life-threatening disease.

### Data collection

This study was conducted by uniformly trained investigators who undertook the investigation process. During the patient’s treatment, the investigator conducted surveys of the patient and their spouse. During the investigation, the investigator answered the respondents’ questions while filling in the questionnaire. The investigator collected and checked the questionnaire on the spot and promptly communicated with the patient to fill in the missing items.

A total of 227 pairs of patients (all female) and their spouses were recruited, and 11 pairs were excluded based on the exclusion criteria. Finally, 216 pairs of cancer patients and their spouses completed the survey. Two researchers input the data to ensure the accuracy of the data.

### Measurements

#### Baseline variables

The researchers designed the general demographic characteristics questionnaire. Basic information included the age, occupation, the education level of the patient and her spouse. The patient case data included the course of the disease, the clinical stage of the tumor, and the surgical method.

#### Illness perception

The Brief Illness Perception Questionnaire (BIPQ), developed by Broadbent et al. [19] and adjusted by Meiyaqi et al. [20] has been widely used to measure the IP of Chinese patients. The questionnaire consisted of eight items, with each item divided into three dimensions: cognitive representation, emotional illness representation, and illness comprehensibility or coherence of illness. We used 0–10 points, with a total score of 0–80 points. The higher the score, the more patients’ cognition and negative emotions about the disease. The Cronbach’s α of the Chinese vision of this scale was 0.77.

#### Psychosocial adjustment to illness

Psychosocial adjustment was assessed using the Psychosocial Adjustment to Illness Scale (PAIS-SR) developed by Derogatis et al. [21] The Chinese version of PAIS-SR, translated by Yao et al. [22], has been widely used in China. The Chinese version of PAIS-SR contains 44 items and the following seven dimensions: health care orientation, vocational environment, domestic environment, sexual relationship, extended family relationship, social environment, and psychological distress. Each item is scored from 0 to 3 points, with total points from 0 to 132. A total of 0–34, 35–50, and 51–132 indicated mild, moderate, and severe, respectively. The higher the score, the greater the psychological and social adjustment problems. The Cronbach’s α of Chinese visions was 0.872.

### Statistic analysis

SPSS24.0 and AMOS24.0 were used for data analysis. The mean and standard deviation were used to describe the measurement data, and the frequency and percentage were used to describe the count data. Chi-square test and paired sample t-test were used to compare patients’ and spouses’ general information, IP, and psychosocial adjustment to illness. The Pearson correlation coefficient was used to test the correlation between variables. When analyzing data between spouses, the two are interdependent, which violates the independence hypothesis of the materiality test [23]. Considering the non-independence of paired samples, Kenny et al. [23] put forward the actor–partner interdependence model (APIM), which has been used in the study of couple interaction. This model is a paired data analysis method mostly used in marriage and family fields and can simultaneously assess the influence between individual variables and between individual and spouse variables [24]. We used a structural equation model (SEM) with maximum likelihood estimation for APIM [25]. Set the calculation formula of k in the software k = p/a, and set clearly what a and p in the model represent respectively. The purpose of calculating the k value is to determine whether the relationship between the patient and the spouse is actor-only, contrast partner, parter-only, or couple partner by comparing the size and confidence interval of the k value between the patient and the spouse, and finally determine the optimal model. And we use the following recommended indicators to judge the fit of the model: χ^2^ /df < 3, GFI > 0.9, AGFI > 0.9, RMSEA < 0.08, SRMR < 0.05, AIC (the lower the AIC value, the better the model fits).

## Results

### Characteristics of the participants

The mean ages of patients and spouses were 52.77 ± 11.04 and 52.70 ± 10.74, respectively. About 53.70% of patients and 38.89% of spouses had an education level at or below junior high school. Most participants had a monthly income of less than 3,000 yuan. According to the clinical data of patients, for 44.44% of patients, the time since diagnosis was within three months. All patients with BC in this study were female. Other characteristics of the samples are shown in Table 1.

**Table 1:** Demographic characteristics of the study population

Variable	Categories	Patient *N*(%)	Spouse *N*(%)
Age		52.77 ± 11.04	52.70 ± 10.74
Education			
	Junior high school and below	116 (53.70)	84 (38.89)
	High school and junior college	52 (24.07)	67 (31.02)
	Bachelor’s degree or above	48 (22.22)	65 (30.09)
Occupation			
	Worker	9 (4.17)	17 (7.87)
	Farmer	56 (25.93)	38 (17.59)
	Self-employed	44 (20.37)	60 (27.78)
	Teachers and civil servants	35 (16.20)	40 (18.52)
	Others	72 (33.33)	61 (28.24)
Individual monthly income (RMB, yuan)			
	Less than 3000	109 (50.46)	87 (40.28)
	3001–5000	48 (22.22)	76 (35.19)
	5001–10,000	50 (23.15)	40 (18.52)
	Above 10,000	9 (4.17)	13 (6.02)
Time since diagnosis			
	0–3 months	96 (44.44)	-
	3–6 months	56 (25.93)	-
	>6 months	64 (29.63)	-
Clinical stage of tumor			
	Period of I	87 (40.28)	-
	Period of II	82(37.96)	-
	Period of III	47(21.76)	-
Treatment Type			
	Surgery only	82(37.96)	-
	Surgery and chemotherapy	87(40.28)	-
	Surgery and radiotherapy	20(9.26)	-
	Surgery, chemotherapy, and radiotherapy	27 (12.50)	-

### IP and PAI scores of patients with breast cancer and their spouses

The total scores of patients’ and spouses’ IP were 57.75 ± 10.91 and 57.10 ± 11.00, and the total scores of PAI were 64.67 ± 6.33 and 64.76 ± 7.49, respectively (Table 2). The total scores of IP and PAI of patients and spouses showed no statistically significant (*p* > 0.05), but the dimensions of emotional illness representation within IP and health care orientation and domestic environment within PAI showed statistically significant differences (*p* < 0.05) (Table 2).

**Table 2:** Illness perception and psychosocial adjustment to illness in patients with breast cancer and their spouses

Variable	Categories	Patient (x ± s)	Spouse (x ± s)	t	*P*-value
Illness perception					
	Cognitive representation	34.45 ± 7.58	35.26 ± 7.32	1.13	0.259
	Emotional illness representation	16.56 ± 3.24	15.53 ± 4.13	2.890	0.004
	Illness comprehensibility or coherence of illness	6.74 ± 2.82	6.31 ± 2.85	1.597	0.111
Total		57.75 ± 10.91	57.10 ± 11.00	0.624	0.533
Psychosocial adjustment to illness					
	Health care orientation	9.08 ± 2.14	8.04 ± 2.42	2.002	0.046
	Vocational environment	10.49 ± 2.67	10.34 ± 2.50	0.594	0.553
	Domestic environment	9.83 ± 2.21	10.40 ± 2.16	2.687	0.007
	Sexual relationship	9.44 ± 2.19	9.80 ± 2.44	1.579	0.115
	Extended relationships	6.75 ± 1.36	6.90 ± 1.58	1.111	0.267
	Social environment	7.88 ± 2.38	7.61 ± 2.33	1.203	0.230
	Psychological distress	11.19 ± 2.10	11.07 ± 2.32	0.543	0.587
Total		64.67 ± 6.33	64.76 ± 7.49	0.146	0.884

### Correlation between IP and PAI

Table 3 shows that the patient’s IP was positively correlated with their PAI and the IP and PAI of their spouses (*p* < 0.01). There was a positive correlation between spouses’ IP and patients’ PAI (*p* < 0.01), as well as between the spouses’ PAI and the patients’ PAI (*p* < 0.01) (Table 3).

**Table 3:** Breast cancer - Correlation of spousal illness perception and psychosocial adjustment to illness

Items	S-IP	*P*-IP	S-PAI	*P*-PAI	S-CR	*P*-CR	S-EIR	*P*-EIR	S-ICC	*P*-ICC
S-IP	1									
P-IP	0.401**	1								
S-PAI	0.177**	0.201**	1							
P-PAI	0.290**	0.379**	0.358**	1						
S-CR	0.910**	0.384**	0.140*	0.209**	1					
P-CR	0.412**	0.922**	0.187**	0.310**	0.410**	1				
S-EIR	0.665**	0.196**	0.054	0.199**	0.388**	0.199**	1			
P-EIR	0.167*	0.629**	0.084	0.255**	0.117	0.366**	0.213**	1		
S-ICC	0.560**	0.279**	0.247**	0.298**	0.385**	0.250**	0.122	0.036	1	
P-ICC	0.251**	0.666**	0.178**	0.340**	0.248**	0.459**	-0.023	0.298**	0.367**	1

Note. S-IP: Spouse-Illness Perception; P-IP: Patient-Illness Perception; P-PAI: Patient-Psychosocial Adjustment to Illness; S-PAI: Spouse-Psychosocial Adjustment to Illness; S-CR: Spouse-Cognitive representation; P-CR: Patient-Cognitive representation; S-EIR: Spouse-Emotional illness representation; P-EIR: Patient-Emotional illness representation; S-ICC: Spouse-Illness comprehensibility or coherence of illness; P-ICC: Patient-Illness comprehensibility or coherence of illness. **p < 0.01, *p < 0.05

### Breast cancer - APIM fitting of IP and PAI in spouses

As patients and spouses can be distinguished by their roles, the standard model of APIM was first tested for distinguishable pairwise relationships (MODEL 1). The results of the actor effect showed that the wife’s IP significantly influenced her own PAI (*β* = 0.182, *p* = 0.001). However, the spouse’s IP did not significantly affect t his own PAI (*β* = 0.078, *p* = 0.113). Regarding the partner effect, the spouse’s IP significantly impacted the patient’s PAI (*β* = 0.095, *p* = 0.015), and the patient’s IP also significantly impacted the spouse’s PAI (*β* = 0.106, *p* = 0.033).

Before calculating the value of k (k = p/a), limiting the actor effect between couples to equal the partner effect did not worsen MODEL 2: χ^2^ = 3.390, *p* = 0.184. The p-value was less than the 0.20 as suggested by Kenny et al. [23], indicating that the model could not accept equal effects for patients and spouses. Therefore, the pairwise model analysis was still calculated as a distinguishable pairwise relationship.

**Fig. 2:**
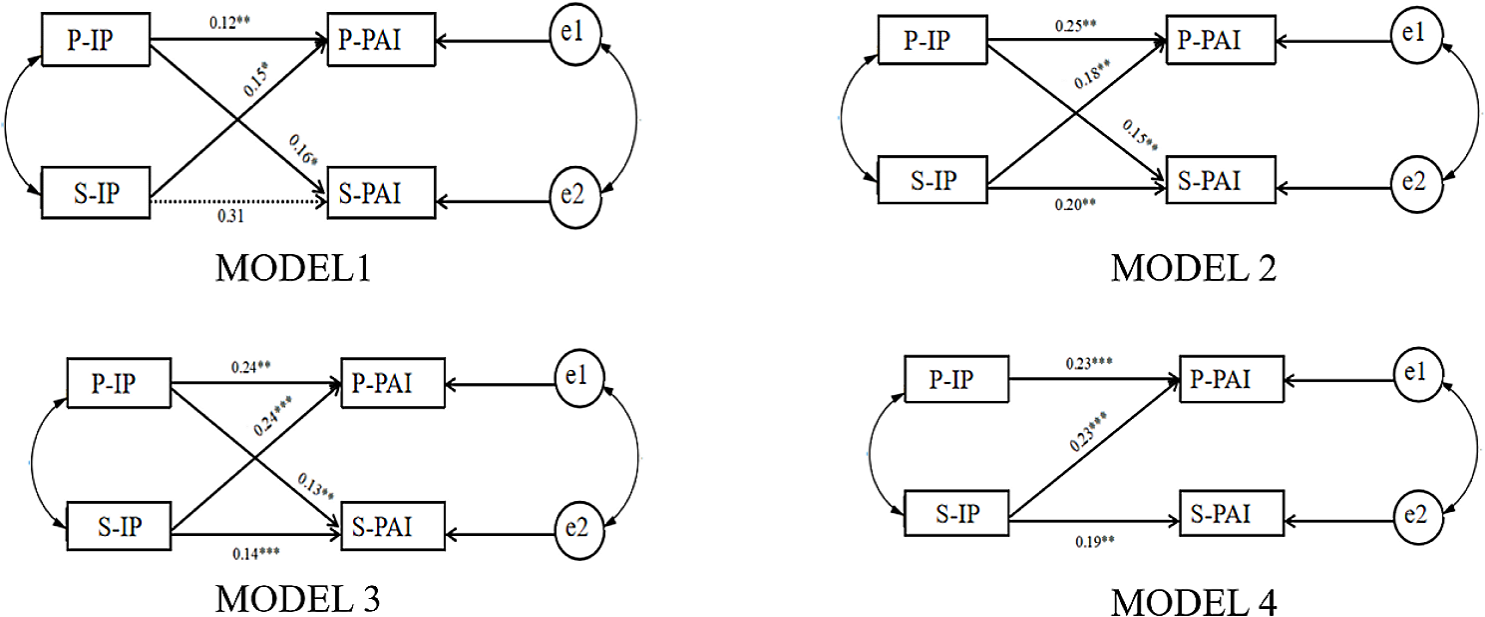
APIM paths of the patients and spouses 1. *Note.* P-IP: Patient-Illness Perception; S-IP: Spouse-Illness Perception; P-PAI: Patient-Psychosocial Adjustment to Illness; S-PAI: Spouse-Psychosocial Adjustment to Illness. *p < 0.05; **p < 0.01; ***p < 0.001

According to the results of the saturation model, the normalized absolute values of the actor effect of the patient and spouse both exceeded 0.1, so the k-value can be estimated by estimating the APIM containing the ghost variable. The confidence interval was determined through 5,000 repeated Bootstrap samplings. After testing, the patient’s k-value (k2) was 0.522, and the 95% confidence interval ranged from 0.058 to 1.329; the confidence interval contained 1, indicating that the patient’s pairing pattern was couple pattern. The spouse’s k-value (k1) was 1.356, and the 95% confidence interval ranged from − 0.068 to 14.187, with 0 and 1 in the confidence interval, indicating that the spouse’s pair pattern was actor-only or couple pattern. To verify the pairing pattern of the spouse and patient, the restriction k was equal to a special value in the confidence interval (1) MODEL 3: k1 = 1, k2 = 1, that is, a1 = p1, a2 = p2; MODEL 4: k1 = 0, k2 = 1, that is, p1 = 0, a2 = p2. The results showed that Model 3 had the best fitting effect, and the patient and spouse were couple pattern (Fig. 2; Tables 4 and 5).

**Table 4:** APIM fitting of breast cancer patient-spouse illness perception and psychosocial adjustment

Model Fit	MODEL 1	MODEL 2	MODEL 3	MODEL 4
χ^2^	-	3.390	1.743	5.151
df	-	2	2	2
P	-	0.184	0.418	0.076
χ^2^/df	-	1.695	0.871	2.575
GFI	1	0.992	0.996	0.988
AGFI	-	0.961	0.980	0.941
RMSEA	-	0.057	0.000	0.086
SRMR	0.000	0.034	0.021	0.046
AIC	20.000	19.390	17.743	21.151

Note. SE: Standardized Estimate; GFI: Goodness of Fit Index; AGFI: Adjusted Goodness of Fit Index; RMSEA: Root mean square error of approximation; SRMR: Standardized root mean square residual; AIC: Akaike Information Criterion

**Table 5:** Effect values for each path in Models 1–4

Model	β	SE	*P*	95%CI
MODEL 1				
Actor effect				
P-IP → P-PAI	0.182	0.313	0.001	(0.190, 0.419)
S-IP → S-PAI	0.078	0.115	0.072	(− 0.009, 0.239)
Partner effect				
P-IP → S-PAI	0.106	0.155	0.014	(0.029, 0.269)
S-IP → P-PAI	0.095	0.165	0.001	(0.190, 0.419)
MODEL 2				
Actor effect				
P-IP → P-PAI	0.142	0.249	0.001	(0.158, 0.340)
S-IP → S-PAI	0.142	0.204	0.001	(0.129, 0.274)
Partner effect				
P-IP → S-PAI	0.103	0.146	0.001	(0.060, 0.228)
S-IP → P-PAI	0.103	0.182	0.001	(0.077, 0.283)
MODEL 3				
Actor effect				
P-IP → P-PAI	0.138	0.238	0.000	(0.057, 0.218)
S-IP → S-PAI	0.092	0.135	0.002	(0.169, 0.306)
Partner effect				
P-IP → S-PAI	0.092	0.134	0.002	(0.058, 0.209)
S-IP → P-PAI	0.138	0.240	0.000	(0.172, 0.304)
MODEL 4				
Actor effect				
P-IP → P-PAI	0.131	0.228	0.000	(0.160, 0.295)
S-IP → S-PAI	0.127	0.186	0.003	(0.063, 0.304)
Partner effect				
P-IP → S-PAI	0.000	0.000	-	-
S-IP → P-PAI	0.131	0.230	0.000	(0.163,0.295)

**Fig. 3:**
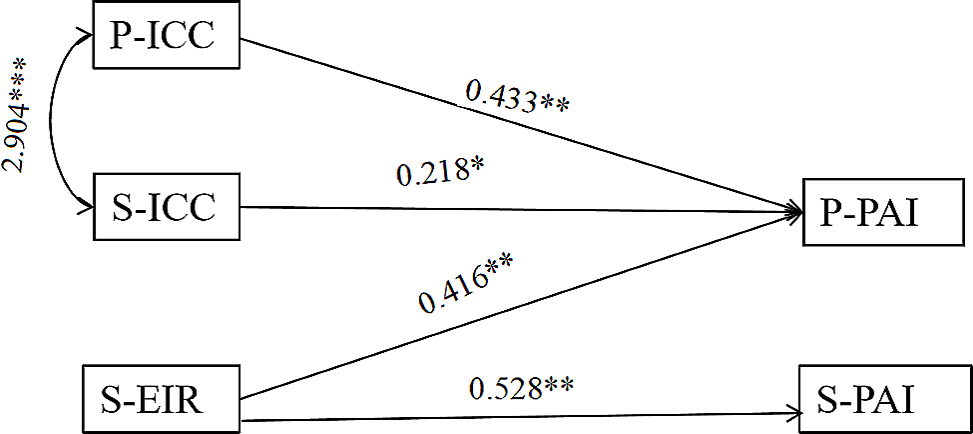
APIM paths of the patients and spouses 2. *Note.* P-PAI: Patient-Psychosocial Adjustment to Illness; S-PAI: Spouse-Psychosocial Adjustment to Illness; S-EIR: Spouse-Emotional illness representation; S-ICC: Spouse-Illness comprehensibility or coherence of illness; P-ICC: Patient-Illness comprehensibility or coherence of illness. ***p < 0.001, **p < 0.01, *p < 0.05

To further explore the relationship between the disease perception and psychosocial adjustment of breast cancer patients and their spouses, the disease perception of patients and spouses was divided into three dimensions: Cognitive Representation, Emotional Illness Representation, and Illness Comprehensibility or Coherence of illness for APIM analysis. The results show that the model fits well, χ^2^ /df = 1.931, GFI = 0.991, AGFI = 0.920, RMSEA = 0.066, SRMR = 0.034, The patient’s psychosocial adjustment to illness is related to the patient’s illness comprehensibility or coherence of illness, the spouse’s emotional illness representation, and the spouse’s illness comprehensibility or coherence of illness, while the spouse’s psychosocial adjustment to illness is only related to the spouse’s illness comprehensibility or coherence of illness. As shown in Fig. 3.

## Discussion

This study investigated the status of illness perception and its association with psychosocial adjustment in patients with BC and their spouses. Additionally, this study explored the interactive impact of IP between patients with BC and their spouses on their psychosocial adjustment based on the APIM. This approach further strengthened the understanding of the mechanism of interaction between the two, providing a unique perspective for future experimental research.

The findings showed that the IP scores of patients with BC and their spouses were (57.75 ± 10.91) and (57.10 ± 11.00) points, respectively, which were similar to the results by Zhou Jie et al. [26], but higher than the results of Xu et al. [27] in patients with lung cancer. This difference may be related to gender [28] and disease characteristics. First, the somatic symptoms and psychological stress of patients participating in the study during the treatment of the disease may affect their perception of the disease [29, 30], resulting in a significantly higher score in the emotional dimension of the patients’ illness perception compared to their spouses. Second, studies have shown that women are more focused on health and emotional expression [31, 32], and women are the predominant group for breast cancer, which means that they may exhibit greater awareness of their feelings and may have a stronger perception of the disease due to the change in body image that comes with breast cancer [31, 33]. In addition, differences in educational attainment may also be a factor influencing their perception of the disease due to differences in the educational level of patients and spouses in this study [34, 35].

In this study, the PAI scores of the patients and their spouses were in the stage of severe maladjustment, which was higher than the results of Shen Aomei et al. [36] This may be because the patients and their spouses in this study are mostly farmers or self-employed individuals. When patients initially receive treatment for their illness, both the patients and their spouses have to temporarily leave their work to cope with the illness together, leading to a sudden decrease in family income. Additionally, due to the high cost of cancer treatment and other factors, both patients and spouses bear a significant psychological burden. In addition, we found that the family relationship dimension of the spouses’ PAI was significantly poorer than that of the patients (*p* < 0.05). This finding may be attributed to the spouse needs to assume multiple roles in the family to make up for the instability of the family structure caused by the illness, leading to potential difficulties in the adjustment process while taking on these responsibilities [5]. Therefore, during medical treatment and diagnosis of patients with BC, medical personnel should not only pay attention to the psychosocial status of patients but also extend their attention to the psychosocial status of spouses. Targeted psychological counseling should be provided to alleviate the psychological stress of both patients and spouses, enhance their ability to cope with and adjust to the disease, and ultimately improve the patients’ quality of life.

Similar to previous studies, we found that patients’ perception of illness was significantly correlated with the level of psychosocial adjustment to illness [37]. Positive perception of illness can improve patients’ self-efficacy and self-management ability, promote emotional and psychosocial adjustment, and improve their quality of life [38, 39]. This study also confirmed the positive relationship between the two: the lower the patient’s perception of illness, the better the psychosocial adjustment. Because the trajectories of couples are often closely related after BC diagnosis [40], they experience a series of BC-associated stresses, such as changes in sexual behavior, during which individual interactions may affect their adjustment to the disease [41]. Moreover, APIM analysis results confirmed that IP and PAI have actor–partner effects in patients with BC and their spouses and formed a couple pattern. Specifically, patients’ PAI was affected by themselves and their spouses, but spouses’ PAI was affected only by their patients. This can be easily comprehended because BC affects both spouses collectively, and a spouse’s BC may bring about changes in the relationship dynamics—either fostering greater closeness or creating distance—and affect the psychosocial status of both partners [15, 42]. However, the PAI of spouses is only influenced by the IP of patients, which further elucidates the specific pathway of the role of IP and PAI between spouses. This finding could be promising as it offers a new perspective and intervention pathway for enhancing PAI in BC patients. For example, IP-related interventions targeted at spouses can enhance the PAI of patients, while IP-related interventions targeted at patients can simultaneously improve the PAI of both spouses. Further research has revealed that the emotional illness manifestation of spouses and the comprehensibility or coherence of the illness can affect the patient’s level of psychosocial adaptation. This underscores the strong interaction between patients and spouses as an intimate unit, where enhancing the patient’s level of psychosocial adaptation can be achieved through intervening in the emotions of the spouse and their understanding of the illness.

### Study limitations

There are some limitations in this study. First, the study only collected data on patients with BC and their spouses at two hospitals, so caution is needed when generalizing the results to other groups. Second, the study used a cross-sectional study design with limited explanations for causality. Therefore, longitudinal studies, including the period from cancer diagnosis to treatment and recovery, are needed along with consideration of the effects of confounding variables on psychosocial adjustment to the disease. Third, these instruments have been validated for use in breast cancer patients, but have not yet been validated in their partners. Fourth, while this study has revealed factors influencing PAI, addressing other factors affecting marital relationships (such as intimacy, dynamics of couples before and after cancer) is necessary.

## Conclusion and clinical implications

In this study, the level of IP and PAI were investigated on 216 pairs of patients with BC and their spouses. The results showed that the PAI of patients with BC was affected by the actor’s IP and the partner’s IP. Therefore, in the future, after the diagnosis of BC disease, medical personnel can implement early psychological intervention by treating patients and their spouses as dual subjects using mindfulness-based stress reduction, cognitive behavioral therapy, and other measures to promote PAI.

## Data Availability

The data is not publicly available because further research is being conducted and more manuscripts are being prepared. Data for the present study will be made accessible upon reasonable request from the principal investigator or corresponding author.
